# Hydrogenation of CO_2_ at ambient pressure catalyzed by a highly active thermostable biocatalyst

**DOI:** 10.1186/s13068-018-1236-3

**Published:** 2018-09-01

**Authors:** Fabian M. Schwarz, Kai Schuchmann, Volker Müller

**Affiliations:** Molecular Microbiology & Bioenergetics, Institute of Molecular Biosciences, Johann Wolfgang Goethe University, Max-von-Laue-Str. 9, 60438 Frankfurt, Germany

**Keywords:** Hydrogen production, Hydrogen storage, Carbon capture, Biohydrogen, Formate dehydrogenase, Hydrogenase, Hydrogen-dependent CO_2_ reductase, Thermophiles, *Thermoanaerobacter kivui*

## Abstract

**Background:**

Replacing fossil fuels as energy carrier requires alternatives that combine sustainable production, high volumetric energy density, easy and fast refueling for mobile applications, and preferably low risk of hazard. Molecular hydrogen (H_2_) has been considered as promising alternative; however, practical application is struggling because of the low volumetric energy density and the explosion hazard when stored in large amounts. One way to overcome these limitations is the transient conversion of H_2_ into other chemicals with increased volumetric energy density and lower risk hazard, for example so-called liquid organic hydrogen carriers such as formic acid/formate that is obtained by hydrogenation of CO_2_. Many homogenous and heterogenous chemical catalysts have been described in the past years, however, often requiring high pressures and temperatures. Recently, the first biocatalyst for this reaction has been described opening the route to a biotechnological alternative for this conversion.

**Results:**

The hydrogen-dependent CO_2_ reductase (HDCR) is a highly active biocatalyst for storing H_2_ in the form of formic acid/formate by reversibly catalyzing the hydrogenation of CO_2_. We report the identification, isolation, and characterization of the first thermostable HDCR operating at temperatures up to 70 °C. The enzyme was isolated from the thermophilic acetogenic bacterium *Thermoanaerobacter kivui* and displays exceptionally high activities in both reaction directions, substantially exceeding known chemical catalysts. CO_2_ hydrogenation is catalyzed at mild conditions with a turnover frequency of 9,556,000 h^−1^ (specific activity of 900 µmol formate min^−1^ mg^−1^) and the reverse reaction, H_2_ + CO_2_ release from formate, is catalyzed with a turnover frequency of 9,892,000 h^−1^ (930 µmol H_2_ min^−1^ mg^−1^). The HDCR of *T. kivui* consists of a [FeFe] hydrogenase subunit putatively coupled to a tungsten-dependent CO_2_ reductase/formate dehydrogenase subunit by an array of iron–sulfur clusters.

**Conclusions:**

The discovery of the first thermostable HDCR provides a promising biological alternative for a chemically challenging reaction and might serve as model for the better understanding of catalysts able to efficiently reduce CO_2_. The catalytic activity for reversible CO_2_ hydrogenation of this enzyme is the highest activity known for bio- and chemical catalysts and requiring only ambient temperatures and pressures. The thermostability provides more flexibility regarding the process parameters for a biotechnological application.

**Electronic supplementary material:**

The online version of this article (10.1186/s13068-018-1236-3) contains supplementary material, which is available to authorized users.

## Background

Conversion of CO_2_ into other chemicals has gained increased attention in recent times primarily caused by the problem of increasing atmospheric CO_2_ concentrations caused by anthropogenic emissions and the need for sustainable technologies for energy conversion and storage, carbon sequestration, as well as chemical production [1]. Among the possible reactions, direct hydrogenation of CO_2_ to formic acid or the conjugate base formate is of special interest. Formate can serve as substrate for production of other carbon containing products by chemical or biotechnological processes or utilized directly for leather processing, food preservative, or as deicing agent [2, 3]. As substrate for biological fermentations, it overcomes mass transfer limitations of gaseous substrates such as synthesis gas [4]. In addition, formate can be utilized as hydrogen storage system coupled to downstream hydrogen release or serve directly as electron source for a fuel cell device [5, 6]. Replacing fossil fuels as energy carrier requires alternatives that combine sustainable production, high volumetric energy density, easy and fast refueling for mobile applications, and preferably low risk of hazard. Molecular hydrogen (H_2_) has been considered as promising alternative technologies either for mobile applications or to store excess electricity generated at off-peak times by wind or solar power. However, the practical application is struggling because of the low volumetric energy density and the explosion hazard when stored in large amounts [7, 8]. One way to overcome these limitations is the transient conversion of H_2_ into other chemicals, for example so-called liquid organic hydrogen carriers (LOHC), with elevated volumetric energy density. Among the multiple technologies envisioned for overcoming this problem, CO_2_ hydrogenation to formic acid represents a promising solution [7, 9]. Formic acid combines its liquid nature with low toxicity and easy handling, prerequisites for the storage and further utilization.

The thermodynamic stability of CO_2_ makes hydrogenation a challenging reaction. Increased attention to this conversion in the last years has led to important advances. Homogenous or heterogenous chemical catalysts have been developed for CO_2_ hydrogenation and studied under multiple conditions [3, 9, 10]. However, many solutions suffer from low turnover frequencies (TOF), dependency on high pressure or temperature, or very expensive additives emphasizing the need for the discovery of better catalysts and a fundamental understanding of the process.

**Fig. 1:**
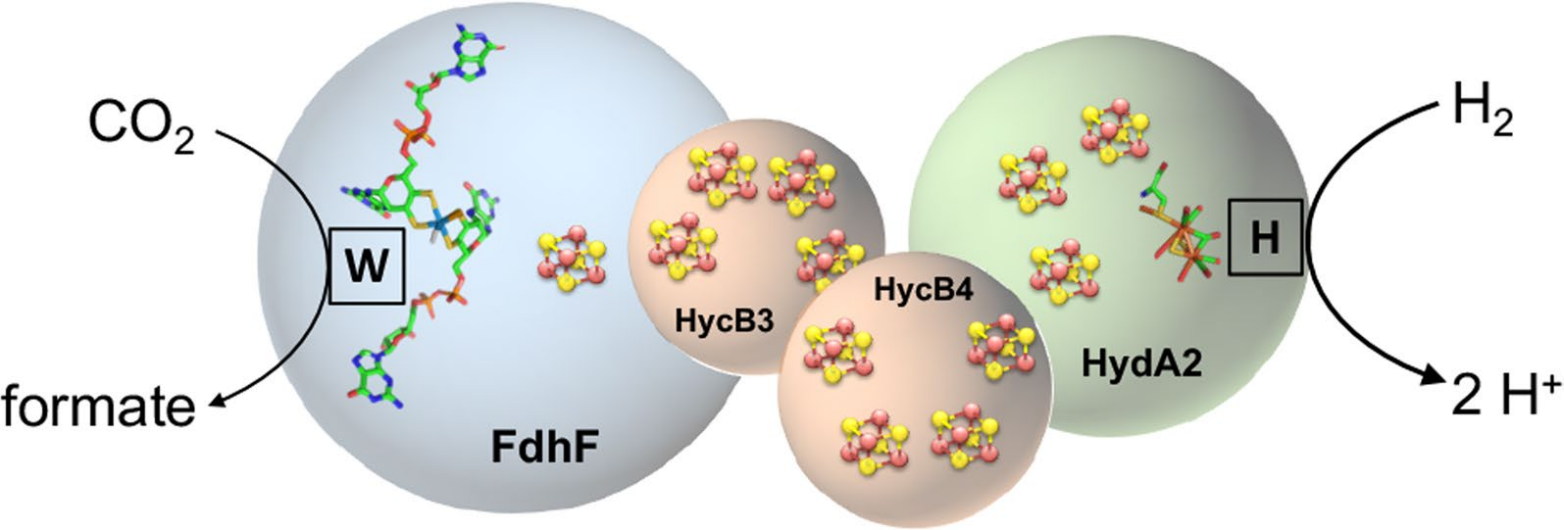
Schematic model of the hydrogen-dependent CO_2_ reductase of *T. kivui*. The enzyme complex consists of a [FeFe] hydrogenase subunit coupled to a metal-containing formate dehydrogenase subunit of the W-*bis* PGD super-family reductase family. Coupling is putatively achieved by two iron–sulfur cluster rich subunits. The model is based on the amino acid sequence of the protein

In contrast to the large number of chemical catalysts, recently the first biocatalyst for direct and reversible hydrogenation of CO_2_ has been described. It operates under ambient pressures and temperatures with a TOF of 101,600 h^−1^ which is significantly better than chemical catalysts [10–12]. The enzyme, named hydrogen-dependent CO_2_ reductase (HDCR), consists of four subunits with a [FeFe] hydrogenase, a molybdenum/tungsten-bis pyranopterin guanosine dinucleotide (bis PGD) cofactor containing formate dehydrogenase and iron–sulfur cluster containing electron transferring subunits (Fig. 1). In contrast to the membrane-bound formate hydrogenlyase (FHL) of *E. coli*, a long well-known enzyme that also connects the electron carriers formate/CO_2_ and H_2_, the HDCR is a soluble cytoplasmic enzyme complex [13–15]. FHL has the physiological function of formate oxidation, explaining its strong bias in catalyzing this direction of the reaction, whereas the HDCR of *A. woodii* showed very similar catalytic rates in both directions [11, 16]. By using catalytic protein film electrochemistry, the hydrogenase subunit was characterized as first completely CO-tolerant [FeFe] hydrogenase with the highest apparent affinity for H_2_ ever reported for a [FeFe] hydrogenase [17]. Otherwise, the enzyme showed the typical phenotype of (standard) [FeFe] hydrogenases. The formate dehydrogenase subunit shows, based on the primary sequence, high similarity to other metal-containing formate dehydrogenases, raising the question why this enzyme is very active towards CO_2_ reduction, whereas many closely related enzymes such as the formate dehydrogenase subunit of the FHL are strongly biased for formate oxidation [18]. In addition to the catalytic properties, the HDCR showed the surprising ability to polymerize into large filamentous structures (around 0.2 µm in length) [19].

The exceptional TOF of the first described HDCR together with the questions raised by this discovery led to the motivation to search for other enzymes of this class with potentially superior catalytic properties and/or increased stability. We focused our search on enzymes from thermophilic sources. The increased temperature should be connected with increased conversions rates, and the adaptation to high temperatures could result in increased stability of the enzyme, both desirable properties for industrial application. So far, the number of known HDCR gene clusters is very limited, especially in thermophilic organisms. However, we could identify a putative gene cluster coding for a thermophilic HDCR in the bacterium *Thermoanaerobacter kivui*. This organism belongs to the group of the acetogenic bacteria as *A.* *woodii* but has a growth optimum at 66 °C [20–23]. *T. kivui* can utilize among others H_2_ + CO_2_, CO, formate, pyruvate or glucose as growth substrate. This organism utilizes the reductive acetyl-CoA pathway (Wood–Ljungdahl pathway) for reduction of two molecules of CO_2_ to acetyl-CoA that is converted to acetate as major end product [20, 21]. In this study, we were successful in isolating the HDCR from *T. kivui*, demonstrating that this first thermostable enzyme for direct hydrogenation of CO_2_ shows remarkable catalytic properties with TOFs of 9,556,000 h^−1^ at 1 bar H_2_ + CO_2_ at 60 °C. Even at 30 °C, the enzyme from *T. kivui* shows an 18-fold higher conversion rate compared to the only other characterized HDCR from *A. woodii*. The enzymes seem to be responsible for the first step of the reductive acetyl-CoA pathway of *T. kivui* catalyzing the essential step of CO_2_ reduction to formate.

## Results and discussion

**Fig. 2:**
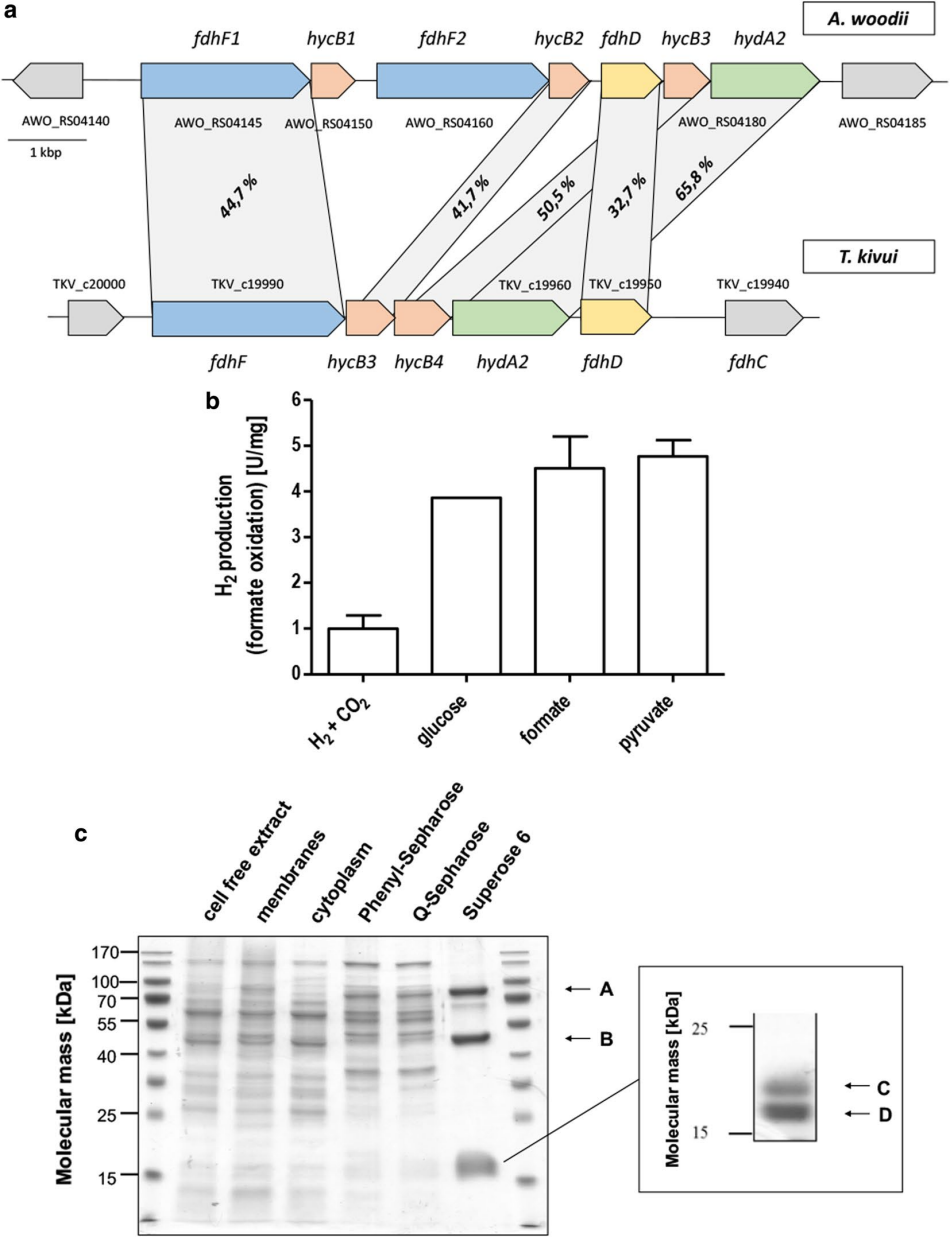
Identification and isolation of the thermostable HDCR. **a** The putative HDCR gene cluster of *T. kivui* compared to the HDCR gene cluster of *A. woodii*. Shown is the present identity of pairwise alignment of the deduced amino acid sequence. For *hyc* genes, only pairs showing highest percent identity are shown. **b** Hydrogen production from formate catalyzed by cell free extracts of *T. kivui* grown on different substrates. 50 mM formate was added to 0.5 mg cell free extract incubated at 60 °C in anoxic buffer (100 mM HEPES/NaOH, 2 mM DTE, pH 7.0) and production of H_2_ was measured in the gas phase. **c** Denaturating SDS polyacrylamide gel electrophoresis of the fractions during isolation of the HDCR from *T. kivui*. 10 µg protein per lane; stained with Coomassie Brilliant blue. Right part, further separation of the small subunits by SDS polyacrylamide gel electrophoresis. LC–MS/MS was used to trace back the isolated protein to its encoding gene; A, fdhF1; B, hydA2; C, hycB2; D, hycB1

To screen for novel HDCRs, we used the gene cluster of the HDCR from *A. woodii* as query to screen available genome sequence data for novel HDCRs. This gene cluster consists of 7 genes containing two isogenes coding for the formate dehydrogenase subunit (fdhF1 and fdhF2), the putative formate dehydrogenase maturation protein fdhD, the [FeFe] hydrogenase hydA2, and three putative electron transferring proteins (hycB1, hycB2, hycB3) (Fig. 2a). Homologs of each gene can be found individually in a multitude of organism making identification of genuine HDCR gene clusters more difficult. Therefore, we used the amino acid sequence of fdhF1, hydA2, hycB1, and hycB3 from *A. woodii* to search for organisms encoding these proteins in their genome using MultiGeneBlast and manually checked these candidates to have the corresponding genes organized in an arrangement resembling the one for *A. woodii*. We identified putative HDCR gene clusters in 25 organisms (Additional file 1: Table S1). A complete HDCR gene cluster was identified in two thermophilic organisms (*Symbiobacterium thermophilum* and *T.* *kivui*). *S. thermophilum* can only grow in coculture with *Geobacillus* sp. [24], we therefore focused on *T. kivui*, a Gram-positive acetogenic bacterium that can grow with H_2_ + CO_2_ as substrates and has a growth optimum of 66 °C [20]. The putative HDCR gene cluster of this organism consists of only 5 genes (Fig. 2a). The two missing genes compared to *A. woodii* are isogenes of the formate dehydrogenase subunit and the corresponding electron transferring Hyc subunit that potentially arose from gene duplication resulting in two variants of the formate dehydrogenase subunit, one with cysteine and one with selenocysteine at the same position, otherwise showing an identity of 80% on the level of the amino acid sequence. In *T. kivui*, only one gene coding for the formate dehydrogenase is present that, based on the primary sequence, does not contain selenocysteine as FhdF2 of *A. woodii*. Two genes hycB1 and hycB2 coding for small putative electron transferring subunits separate the formate dehydrogenase gene from the putative hydrogenase gene hydA2. At the end of the gene cluster, fdhD codes for a putative maturation protein followed by a putative formate transporter fdhC.

To verify the presence of a functional HDCR, *T. kivui* was grown with the substrates H_2_ + CO_2_, glucose, formate or pyruvate. Using cell free extracts of these cells, it could be demonstrated that hydrogen was indeed produced from formate as substrate with specific activities up to 5 µmol H_2_ min^−1^ mg^−1^ (specific hydrogen productivity of 300 mmol H_2_ h^−1^ g^−1^) with cell free extracts from cells grown on formate or pyruvate showing the highest specific activities (Fig. 2b). To isolate the HDCR from *T. kivui*, the organism was grown with pyruvate as substrate because this substrate led to the highest specific hydrogen productivity combined with higher cell yields compared to formate grown cells, both requirements to achieve high enzyme yields for subsequent enzyme characterization and applications. Cells were grown to the late exponential growth phase, harvested and disrupted with a French pressure cell press. Membranes were removed by ultracentrifugation, and the HDCR complex was isolated using anoxic conditions from the cytoplasm by hydrophobic interaction chromatography on Phenyl-Sepharose followed by ion exchange chromatography on Q-Sepharose and gel filtration on Superose 6. Formate oxidation coupled to reduction of methylviologen was used as assay to identify formate dehydrogenase activity in all purification steps. This procedure yielded an apparently homogeneous preparation and the enzyme was purified 55-fold compared to the cell free extract. The isolated enzyme was separated by SDS polyacrylamide gel electrophoresis yielding four different subunits (Fig. 2c). LC–MS/MS identified these subunits as being indeed encoded by the genes of the putative HDCR gene cluster as described before (TKV_c19950–TKV_c19990). The isolated enzyme contained the subunits FdhF, HydA2, HycB3, and HycB4. FdhD and FdhC could not be identified to be part of the enzyme. The calculated cumulative molecular mass of the enzyme complex is 177.3 kDa based on a stoichiometry of α_1_β_1_γ_1_δ_*1*_. We used ICP-MS to analyze the metal content of the purified enzyme. Based on the primary sequence, the predicted cofactor content of the entire complex is 12 [4Fe4S] clusters, the di-iron center of the [FeFe] hydrogenase subunit and the bis PGD cofactor of the formate dehydrogenase subunit. We detected 42 mol Fe, < 0.05 mol Mo, 0.34 mol W, 4.5 Zn per mol of enzyme. The iron content is lower than the expected 50 mol Fe per mol of enzyme indicating that during protein purification, part of the cofactors could have been lost. Interestingly, molybdenum could not be detected in the enzyme but tungsten. In contrast to the HDCR from *A. woodii,* the enzyme from *T. kivui* seems to contain a formate dehydrogenase of the tungstoenzyme family and not molybdenum bound to the bis PGD cofactor.

**Fig. 3:**
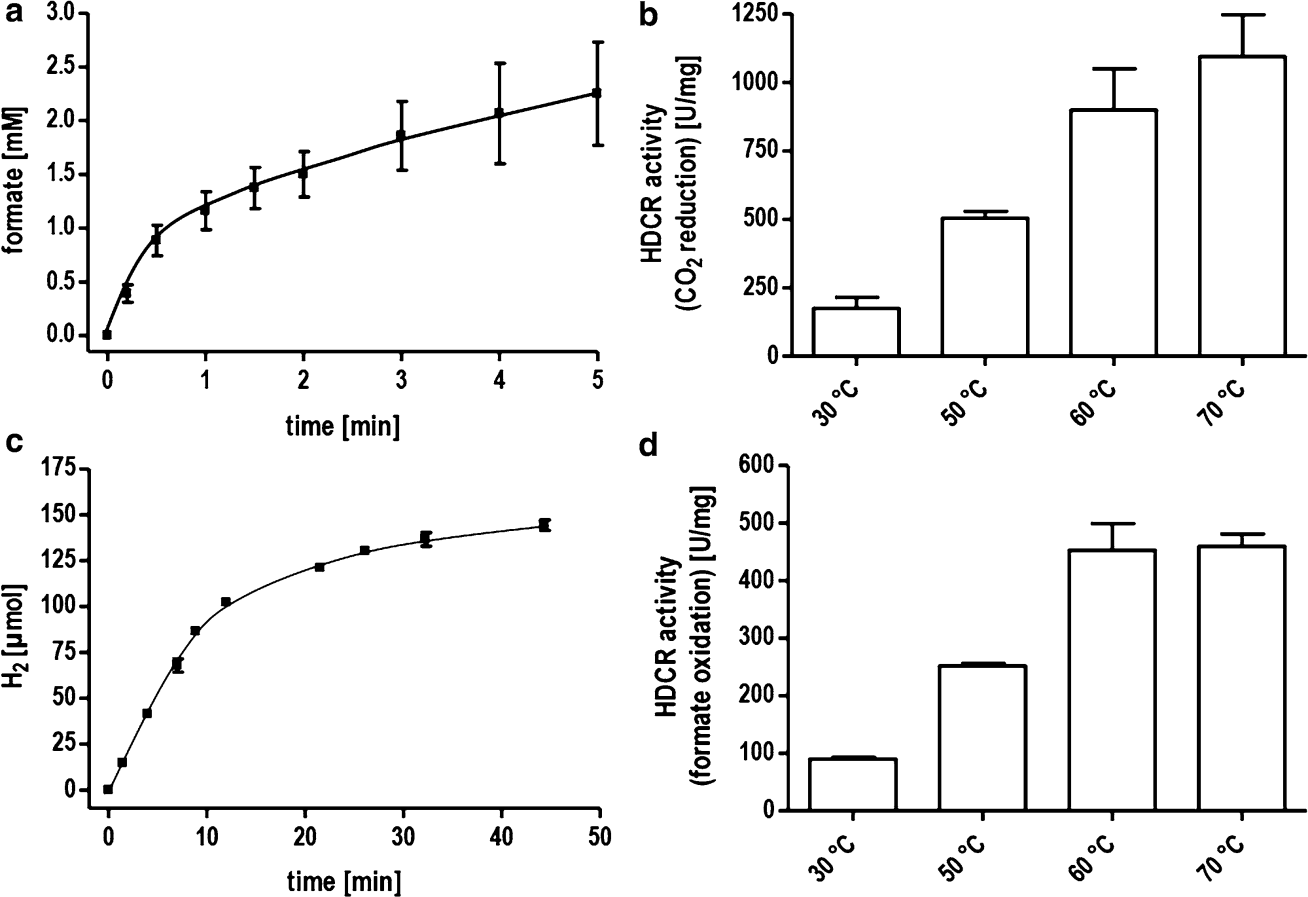
Hydrogenation of CO_2_ and H_2_ evolution from formate catalyzed by the HDCR. **a** and **b** 10 µg isolated enzyme was incubated under an atmosphere of H_2_ + CO_2_ (80:20 [v:v], 1.1 × 10^5^ Pa) in 100 mM HEPES/NaOH, 2 mM DTE, pH 7.0 at 60 °C (**a**) or temperatures as indicated (**b**). **c** and **d** 10 µg isolated enzyme was incubated in 100 mM HEPES/NaOH, 2 mM DTE, pH 7.0 under an atmosphere of 100% N_2_ and with 150 mM (**c**) or 50 mM (**d**) Na formate as substrate at 60 °C (**c**) or temperatures as indicated (**d**). H_2_ production was measured in the gas phase

Next, we wanted to analyze the coupling between hydrogen oxidation and CO_2_ reduction therefore incubated the isolated enzyme under an atmosphere of H_2_ + CO_2_ (80:20 [v:v], 1 bar) at 60 °C. Samples were taken under anoxic conditions and the formate concentration was determined by an enzymatic assay. Formate was produced with an initial turnover frequency of 9,565,000 h^−1^ (specific activity of 900 µmol formate min^−1^ mg^−1^) (Fig. 3a). This rate outcompetes the enzyme from *A. woodii* by a factor of 96 (Additional file 1: Table S2) and is roughly 1200 times faster than chemical catalysts under comparable conditions [25] and almost ten times faster than the best-known chemical catalysts (p(H_2_/CO_2_) 30 bar/10 bar; 120 °C) [9, 11, 26]. The reverse reaction, oxidation of formate to CO_2_ coupled to H_2_ evolution was catalyzed in this assay with a TOF of 4,814,000 h^−1^ (450 µmol H_2_ min^−1^ mg^−1^) (Fig. 3c). Noticeably, we observed using this assay that the HDCR did not show an expected substrate proportionality. By decreasing the amount of enzyme below 10 µg, the specific activity further increased but the catalyst sharply lost the activity below a concentration of 3 µg ml^−1^. Therefore, for stability reasons, we used increased enzyme concentrations for further measurements.

**Fig. 4:**
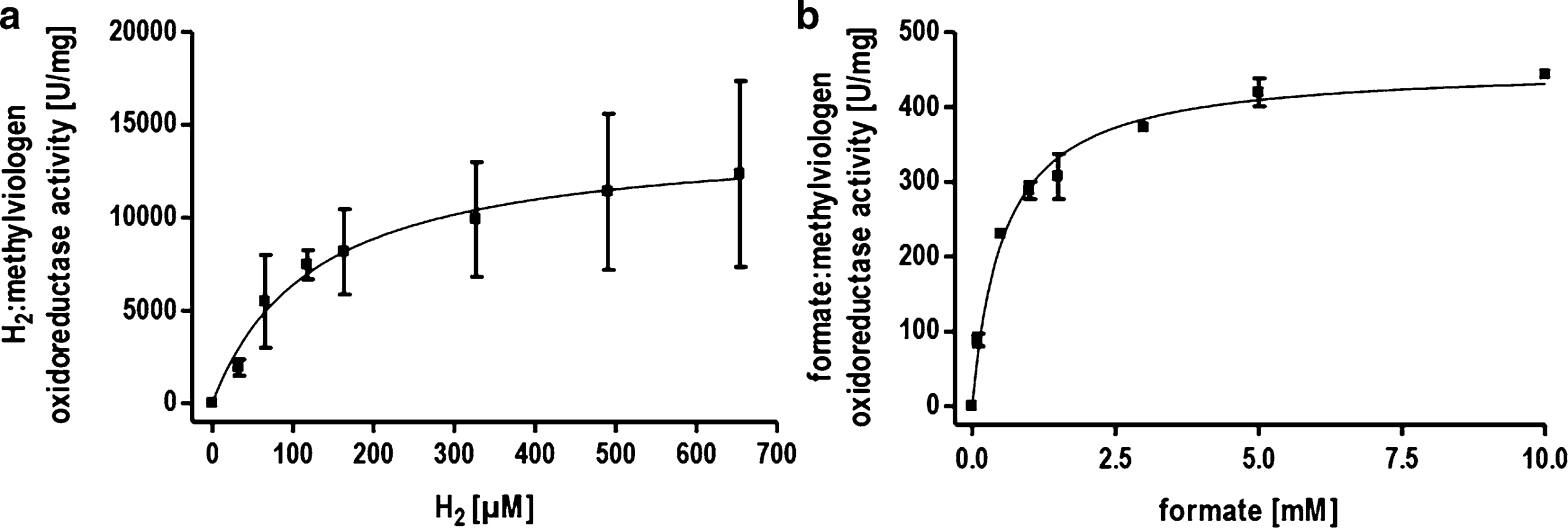
Determination of the *K*_M_ value for formate and H_2_. **a** Methylviologen-dependent hydrogenase activity was measured with H_2_ as electron donor and methylviologen (10 mM) as electron acceptor in 1 ml buffer (100 mM HEPES/NaOH, 2 mM DTE, pH 7.0) and a gas phase of N_2_ and varying amounts of H_2_ at a pressure of 1.1 × 10^5^ Pa. **b** Methylviologen-dependent formate dehydrogenase activity was measured as described for **a** except that the gas phase was 100% N_2_ and varying concentrations of formate were used as electron donor

**Fig. 5:**
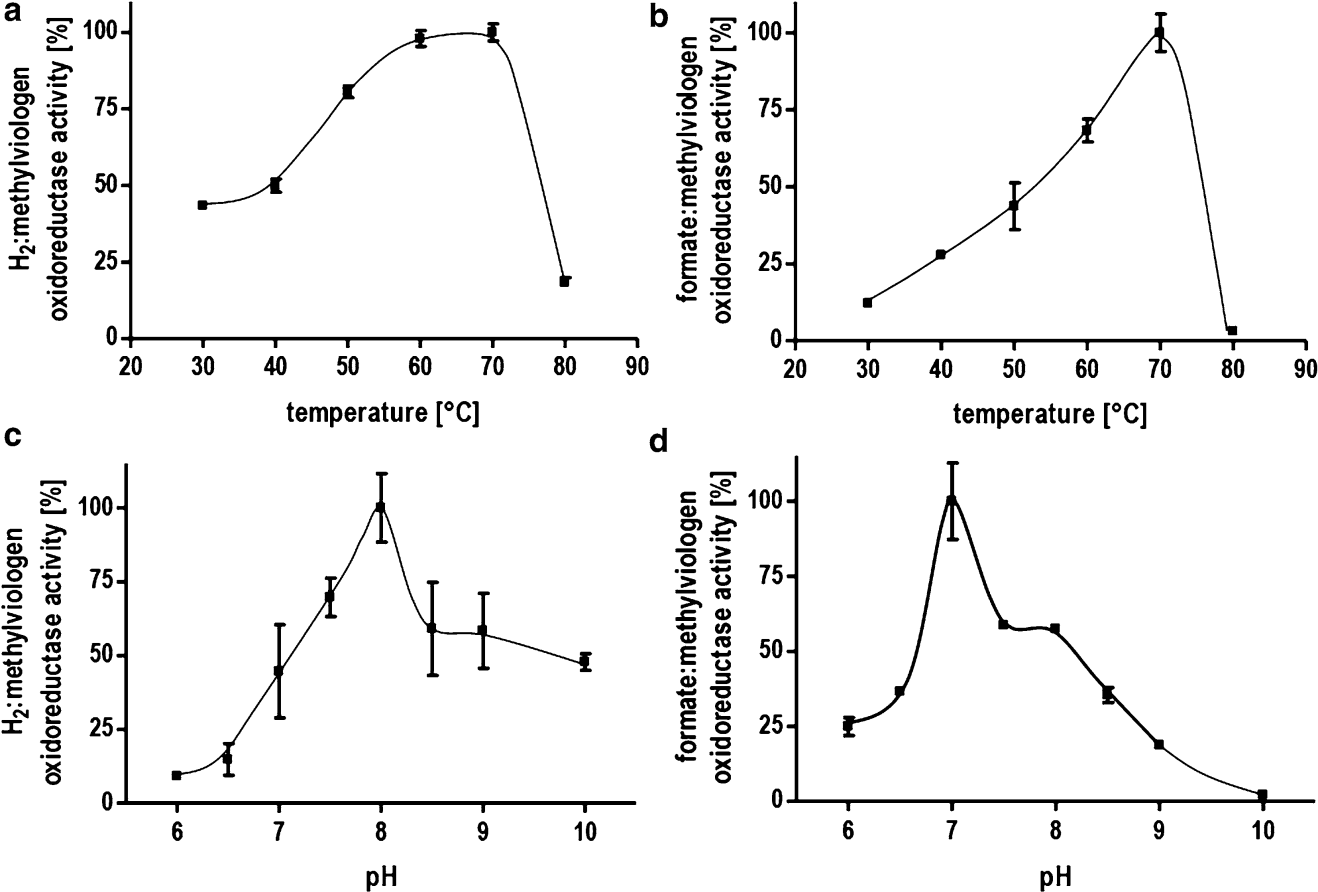
Characterization of the enzymatic properties of the HDCR from *T. kivui*. Methylviologen-dependent hydrogenase and formate dehydrogenase activity was measured with H_2_ (100% in the gas phase, 1.1 × 10^5^ Pa) or formate (10 mM) as electron donor and methylviologen (10 mM) as electron acceptor. **a** Temperature dependence of the hydrogenase activity. **b** Temperature dependence of the formate dehydrogenase activity. **c** pH dependence of the hydrogenase activity. **d** pH dependence of the formate dehydrogenase activity. **a** and **b** buffer: 100 mM HEPES/NaOH, 2 mM DTE, pH 7.0. **c** and **d** buffer: 50 mM MES, 50 mM MOPS, 50 mM HEPES, 50 mM EPPS, 50 mM CHES, 2 mM DTE, pH as indicated

To analyze the catalytic properties of part activities of the HDCR, we utilized methylviologen as artificial electron acceptor and formate or H_2_ as electron donor with the isolated HDCR as catalyst. Reduction of methylviologen was followed with UV/Vis spectrophotometry at 604 nm. Correlation of enzyme activity and hydrogen concentration followed a typical Michaelis–Menten kinetic with an apparent K_M_ for H_2_ of 0.13 mM (Fig. 4a). This value is lower than K_M_ values reported for other typical [FeFe] hydrogenases [17, 27]. The specific activity for H_2_ oxidation was 14,400 µmol H_2_ min^−1^ mg^−1^ (TOF 42,266 s^−1^). The formate oxidation showed an apparent Michaelis–Menten kinetic at low formate with an apparent K_M_ of 0.55 mM for formate and a specific activity for formate oxidation of 455 µmol formate min^−1^ mg^−1^ (TOF 1335 s^−1^) (Fig. 4b). This result does not apply to high formate concentrations as will be shown later. The most striking feature of the novel HDCR isolated from *T. kivui* is its thermostability. Higher temperatures are typically connected to higher catalytic rates and provide process advantages especially considering whole cell catalysis. Higher temperatures reduce the energy costs necessary to cool down the fermentation and decrease the risk of contamination. The thermostability of the isolated HDCR was tested first using the artificial electron acceptor methylviologen to study the part reactions separately. The hydrogenase activity showed a maximum at 70 °C and decreased drastically above this temperature (Fig. 5a). Decreasing the temperature had a less severe effect with almost 50% of the maximum activity still present at 30 °C. The formate dehydrogenase activity also showed a maximum at 70 °C but a steeper decrease in activity at lower temperatures with only 10% of the maximum activity present at 30 °C (Fig. 5b). The coupled activity, hydrogen-dependent reduction of CO_2_ showed the highest TOF at 70 °C (11,623,000 h^−1^) (Fig. 3b). Surprisingly, at 30 °C, the TOF was still 1,856,000 h^−1^. This rate is 18 times faster than the one observed for the mesophilic *A. woodii* enzyme with an optimum at 30 °C. The same was true for the reverse reaction, H_2_ production from formate (Fig. 3d). Next, the optimum pH range for catalysis was determined. The activity profiles differed for the part activities with a pH optimum for hydrogen oxidation at a pH of 8 and an optimum of pH 7 for the formate dehydrogenase (Fig. 5c, d). However, at pH 7, the hydrogenase activity is only at 50% of its maximum but in absolute terms still one order of magnitude faster than the formate dehydrogenase activity thus not limiting the coupled reaction.

The HDCR of *T. kivui* is not only the fastest catalyst known for CO_2_ hydrogenation, but as shown before also for the reverse reaction, hydrogen evolution from formate. We wanted to analyze the maximum formate concentrations that can be used before inhibitory effects reduce the catalytic rates. We tested concentrations up to 1M of sodium formate. To our surprise, increasing the substrate concentrations did not only show no inhibition of the enzyme but increased the activity significantly (Additional file 1: Figure S1). The tested substrate concentrations are over 2 orders of magnitude above the *K*_M_ value; therefore, the increase in activity must result from other effects, potentially a second low affinity formate binding site. At 1M sodium formate, hydrogen was produced with an activity of 650 µmol H_2_ min^−1^ mg^−1^ (TOF 6,940,000 h^−1^). As a control if the effect stems from the increasing salt concentration, we tested 50 mM sodium formate with 950 mM sodium chloride but this did not result in increased activity. We also observed a sharply increasing specific activity when using enzyme concentrations below 6 µg ml^−1^ as described before at constant formate concentrations of 150 mM. The maximum activity measured at 3 µg ml^−1^ was 930 µmol H_2_ min^−1^ mg^−1^ (TOF 9,892,000 h^−1^). However, decreasing the concentration further resulted in an immediate total loss of activity (data not shown). We can only speculate that this unusual behavior of the enzyme is connected to the quaternary structure. For the *A. woodii* enzyme, it has been described that the enzyme polymerizes in the presence of divalent cations in vitro into filamentous structures of up to 0.2 µm in length and that this behavior is connected to the enzyme activity. Polymerization of the *T. kivui* enzyme into high molecular weight structures was indicated by the observed elution volume of the isolated HDCR on the gelfiltration resembling the behavior of the *A. woodii* enzyme.

**Fig. 6:**
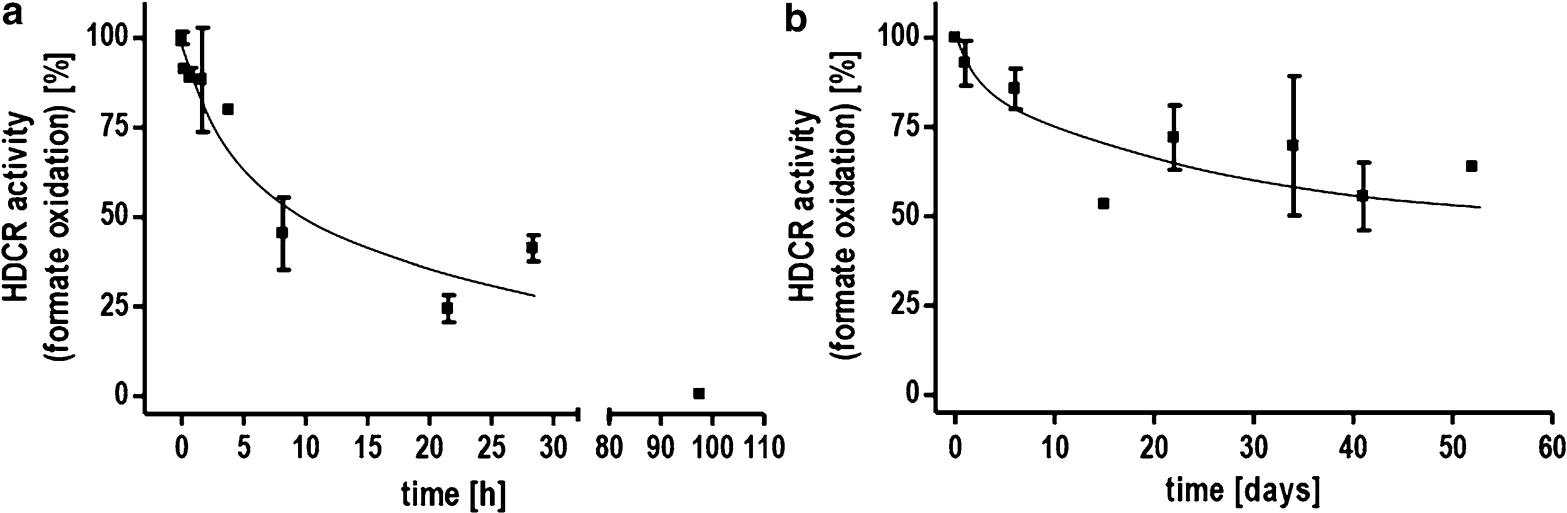
Stability of the HDCR from *T. kivui*. The enzyme was incubated at 60 °C (**a**) and room temperature (**b**). Samples were taken and the specific activity determined by measuring H_2_ evolution. 10 µg isolated enzyme was incubated in 100 mM HEPES/NaOH, 2 mM DTE, pH 7.0 under an atmosphere of 100% N_2_ and 150 mM Na formate as substrate. H_2_ production was measured in the gas phase

For application of the isolated enzyme, the stability is a critical factor. The enzyme stability was tested by incubating the isolated enzyme at two different temperatures and determining the specific activity over time. At 60 °C, the activity dropped to 50% of the initial activity after 9 h (Fig. 6b). Afterwards, the decrease was slower with 25% of the activity still retained after 34 h. At room temperature, 54% of the initial activity was still present after over 52 days. This is worth paying attention since as reported before, at 30 °C the HDCR of *T. kivui* is still much faster than the best-known catalyst at this temperature so far. For storage, we tested storing the enzyme at 4 °C and using repeated freezing (− 21 °C) and thawing cycles. The enzyme was not strongly affected by freezing and retained almost 80% of the initial activity after 52 days and 8 freezing/thawing cycles (Additional file 1: Figure S2). Storage at 4 °C led to a decrease to 50% of the activity after 27 days.

## Conclusions

In summary, the HDCR from *T. kivui* is the first description of a thermostable member of this new enzyme class of CO_2_ reductases that are directly coupled to hydrogen oxidation. The enzyme was isolated from a thermophilic organism that has a growth optimum at 66 °C and showed a similar temperature optimum in vitro of 70 °C. The enzyme is a complex of four subunits, a [FeFe] hydrogenase, a formate dehydrogenase, and two putatively electron transferring subunits connecting the two redox reactions. The enzyme displayed remarkable catalytic properties with a TOF for formate formation from hydrogen and CO_2_ of 9,556,000 h^−1^ and 9,892,000 h^−1^ for the reverse reaction. This is, in both directions, significantly faster than state-of-the art chemical catalysts while needing only ambient pressure and moderate temperatures. The novel HDCR is also superior to biological catalysts described for this reaction and the thermostability as well as the broad temperature range for activity offers more flexibility for process development (Additional file 1: Table S2). Given the important problem of hydrogen storage, the reversible catalyst described in this study offers a promising step towards the efficient conversion of hydrogen into the liquid organic hydrogen carrier formic acid/formate and the subsequent release of the gas for the following utilization as energy source. The characterization of more highly active CO_2_ reductases will also help to attain a better understanding of the factors required for efficient catalysis. Noticeably, the highly active HDCR enzymes contain a formate dehydrogenase subunit that, based on the primary sequence, does not show an obvious difference from other metal-dependent formate dehydrogenases of the DMSO reductase family of which most show only low activity towards CO_2_ reduction and have a strong bias for formate oxidation. Comparing the two HDCRs described so far, it strikes the attention that the more active enzyme from *T. kivui* does not contain selenocysteine as does the less active *A. woodii* enzyme. The presence of selenocysteine is typically connected to increased catalytic activities due to the lower pK_a_ value and reduction potential of the selenol group compared to the thiol group of cysteine [28, 29]. However, the molybdenum bound to the bis PGD cofactor has been replaced by tungsten in *T. kivui*, a metal often found in highly active CO_2_ reductases and especially in enzymes operating at increased temperatures [30, 31]. To shed more light on the catalysis of the HDCR enzymes a structure of a complete HDCR or the formate dehydrogenase subunit is urgently needed to reveal the architecture of the active site and to identify the factors determining its high activity. This could also help to design more active bio-inspired chemical catalysts, since utilization of CO_2_ as substrate is at the moment obstructed by the availability of efficient catalysts.

## Methods

### Preparation of cell free crude extracts

*Thermoanaerobacter kivui* LKT-1 (DSM 2030) was cultivated in 1-l flasks (Müller-Krempel, Bülach, Switzerland) containing 500 ml of complex medium previously described [21]. The media were prepared using anaerobic techniques as described [32, 33]. A temperature of 66 °C was routinely used for the cultivation. Sterile glucose, formate, or pyruvate was added as carbon source from an anoxic stock solution. Respectively, H_2_ + CO_2_ was used as an electron donor and carbon source. Therefore, 1-l flasks were pressurized with 1 bar H_2_ + CO_2_ (80:20 [v/v]) containing only 250 ml complex medium to increase the gas-to-liquid ratio. Growth was determined by measuring the optical density at 600 nm with a UV/vis spectrophotometer. All buffers used in the following preparation procedure and the subsequent enzyme activity measurements contained 2 mm dithioerythritol and 4 μm resazurin. All steps were performed under strictly anoxic conditions at room temperature in an anaerobic chamber (Coy Laboratory Products, Grass Lake, MI) filled with a gas composition of 95–98% N_2_ and 2–5% H_2_ as described [34]. For the preparation of cell free extracts, the cells were harvested in the late exponential growth phase and were subsequently washed with buffer A (25 mM Tris/HCl, 20 mM MgSO_4_, 20% glycerin [v/v], pH 7.5). Afterwards, the cells were resuspended in 3 ml buffer A including 0.5 mM PMSF and a spade point DNaseI. Cells were disrupted by a single passage through a French pressure cell press (SLM Aminco, SLM Instruments, USA) at 97 MPa. Cell debris was removed by centrifugation at 2000×*g* for 20 min. The supernatant was immediately used for the measurement of the hydrogen production from formate in the way described below.

### Purification of the HDCR complex

Cells of *T. kivui* LKT-1 (DSM 2030) were grown heterotrophically with pyruvate as substrate at 66 °C under anoxic conditions in 20-l flasks (Glasgerätebau Ochs; Bovenden-Lenglern, Germany). All purification steps of the HDCR complex were performed under strictly anoxic conditions at room temperature in an anaerobic chamber as described before. Cells of *T. kivui* were harvested at an OD 600 of around 1.3 and were washed twice with buffer A (25 mM Tris/HCl, 20 mM MgSO_4_, 20% glycerin [v/v], pH 7.5). Afterwards, the cells were resuspended in 15 ml buffer A including 0.5 mM PMSF and a spade point DNaseI. Cells were disrupted by a single passage through a French pressure cell press (SLM Aminco, SLM Instruments, USA) at 110 MPa. Cell debris was removed by centrifugation at 23,700×*g* for 20 min at 4 °C. To separate the membranes from the cytoplasmic fraction, an ultracentrifugation at 184,000×*g* for 45 min was performed. The supernatant containing the cytoplasmic fraction with around 1800 mg of protein was used for the further purification.

Ammonium sulfate (0.4 M) was added to the cytoplasmic fraction and the sample was loaded onto a Phenyl-Sepharose high-performance column (2.6 cm × 6.4 cm) equilibrated with buffer B (25 mM Tris/HCl, 20 mM MgSO_4_, 20% glycerin [v/v], 0.4 M (NH_4_)_2_SO_4_, pH 7.5). Methylviologen-dependent formate dehydrogenase activity eluted at (NH_4_)_2_SO_4_ concentrations below 0.4 M in a linear gradient over 120 ml from 0.4 M to 0 M (NH_4_)_2_SO_4_. The fractions with Methylviologen-dependent formate dehydrogenase activity were pooled and the sample was diluted to a conductivity of 14 mS/cm with buffer A. Then, the sample was applied to a Q-Sepharose high-performance column (1.6 cm × 11.9 cm) equilibrated with buffer A. The formate dehydrogenase activity was found in the flow through. The pooled fractions were concentrated by using ultrafiltration in 100-kDa VIASPIN tubes (Sartorius Stedim Biotech GmbH, Germany). Half of the concentrated sample was loaded on a Superose 6 10/300 GL prepacked column (GE Healthcare Life Sciences, Little Chalfont, UK) equilibrated with buffer C (25 mM Tris/HCl, 20 mM MgSO_4_, 20% glycerin [v/v], 150 mM NaCl, pH 7.5) and eluted at a flow rate of 0.5 ml/min. Formate dehydrogenase activity eluted not in a defined single peak but spread over a wide elution volume. This step was repeated with the second half of the concentrated sample in a separate run to achieve a better separation. The formate dehydrogenase activity was enriched by 55-fold from the cytoplasm resulting in 1.5–2 mg of homogeneous protein from 36 g of wet cell mass. The fractions were pooled and stored at 4 °C.

### Measurement of enzyme activity

All enzyme assays, unless otherwise stated, were performed in 1.8 ml anaerobic cuvettes (Glasgerätebau Ochs, Bovenden-Lenglern, Germany) sealed by rubber stoppers at 60 °C and filled with 1 ml buffer. In all enzyme assays, the buffer was pre-incubated at the temperature of interest.

Methylviologen-dependent formate dehydrogenase activity was measured with formate (10 mM) as electron donor and methylviologen (10 mM) as electron acceptor in 1 ml buffer D (100 mM HEPES/NaOH, 2 mM DTE, pH 7.0) and a gas phase of 100% N_2_ at a pressure of 1.1 × 10^5^ Pa. The reduction of Methylviologen was monitored at 604 nm by UV/Vis spectrophotometry (*ε* = 13.9 mM^−1^ cm^−1^).

Measurements of Methylviologen-dependent hydrogenase activity were performed under the same conditions except that the gas phase was 100% H_2_ at a pressure of 1.1 × 10^5^ Pa. Formate was omitted in the enzyme assay and Methylviologen reduction was measured at 604 nm as described before. For *K*_M_ determination, the H_2_ concentration in the gas phase was manually adjusted using a gas tight syringe. The concentrations of H_2_ in the liquid phase were calculated according to Henry’s law.

For the determination of the pH and temperature profile, the enzyme was preincubated 10 min at the pH or temperature indicated, respectively. The reaction temperature for the determination of the pH optima was set to 60 °C. The buffer used for the pH optima determination was 50 mM MES, 50 mM MOPS, 50 mM HEPES, 50 mM EPPS, 50 mM CHES, 2 mM DTE, and the pH as indicated.

Hydrogen-dependent carbon dioxide reductase activity was measured in a two-step enzyme assay. In the first part, the hydrogen-dependent CO_2_-reduction was started by using a gas phase of 80% H_2_ + 20% CO_2_ [v/v] at a pressure of 1.1 × 10^5^ Pa in 120-ml serum bottles (Glasgerätebau Ochs, Bovenden-Lenglern, Germany) sealed by rubber stoppers, containing 5 ml buffer D at 60 °C in a shaking water bath. At defined time points, a sample was taken from the reaction mixture and stored on ice. Afterwards, the determination of the formate concentration of all samples was carried out by using a commercially available formic acid-kit (Boehringer Mannheim/R-Biopharm AG, Mannheim/Darmstadt, Germany). It contained a formate dehydrogenase as a reporter enzyme and the formation of NADH was monitored at 340 nm.

Hydrogen production from formate was measured in 9-ml serum vials (Glasgerätebau Ochs, Bovenden-Lenglern, Germany) sealed by rubber stoppers, containing 1 ml buffer D at 60 °C in a shaking water bath. The gas phase in the serum vials was 100% N_2_ at atmospheric pressure. Before each gas sample was taken, the overpressure in the serum vials due to the H_2_ production from formate oxidation was determined and adapted to ambient pressure. Gas samples of 50 µl volume were withdrawn with a gas-tight syringe (Hamilton CO. glass syringe, Reno, USA). The concentrations of H_2_ were determined by using a gas chromatograph (Clarus 580 GC; PerkinElmer, Waltham, MA, USA) with a Shin Carbon ST 80/100 column (Restek GmbH, Bad Homburg, Germany) kept at 40 °C. Nitrogen was used as carrier gas, respectively, with a head pressure of 400 kPa and a split flow of 30 ml/s. H_2_ was detected with a thermal conductivity detector kept at 100 °C.

### Analytical methods

The protein concentration was measured according to Bradford [35]. Proteins were separated in 12% polyacrylamide gels and stained with Coomassie brilliant blue G250.

### Bioinformatic methods

All DNA and protein sequences were retrieved from the National Center for Biotechnology Information database. Homology searches were performed using BLASTp with default settings (http://blast.ncbi.nlm.nih.gov/Blast.cgi). Protein sequences were analyzed for conserved domains and functional sites by InterProScan 5 [36]. For pairwise sequence alignment, we used EMBOSS Water using default settings [37]. Novel putative HDCR gene cluster was identified using MultiGeneBlast with the amino acid sequence of FdhF1, HycB1, HycB3, and HydA2 from *A. woodii* as input [38]. Minimal percent identity was set to 30, minimal sequence coverage to 25 and maximum distance of the genes to 20 kB. We searched against all available completed bacterial RefSeq genomes (1543 genomes in total).

### Chemicals

All chemicals were supplied by Sigma-Aldrich Chemie GmbH (Munich, Germany) and Carl Roth GmbH & Co KG (Karlsruhe, Germany). All gases were supplied by Praxair (Düsseldorf, Germany).

## Additional file

**Additional file 1: Table S1.** Putative HDCR gene clusters found in bacterial genomes. **Table S2.** Comparison of purified enzymes catalysing the direct hydrogenation of CO_2_ as well as formate oxidation. **Figure S1.** Substrate tolerance of the HDCR from *T. kivui*. **Figure S2.** Stability of the HDCR from *T. kivui*.
