# Supplementary material for: Hydrogenation of CO2 at ambient pressure catalyzed by a highly active thermostable biocatalyst
Source: Biotechnol Biofuels. 2018 Sep 1;11:237. doi: 10.1186/s13068-018-1236-3 (PMC6119302; doi:10.1186/s13068-018-1236-3)
Supplement: Supplementary file 1 — Additional file 1: Table S1. Putative HDCR gene clusters found in bacterial genomes. Table S2. Comparison of purified enzymes catalysing the direct hydrogenation of CO2 as well as formate oxidation. Figure S1. Substrate tolerance of the HDCR from T. kivui. Figure S2. Stability of the HDCR from T. kivui. [file 13068_2018_1236_MOESM1_ESM.docx]

Table S1. Putative HDCR gene clusters found in bacterial genomes. Subunits of the HDCR from A. woodii were used to search available completed bacterial RefSeq genomes for similar gene clusters using MultiGeneBlast. If variations to the A. woodii gene cluster are present they are indicated.

| Organism | Putative HDCR gene cluster |
| --- | --- |
| *Acetobacterium woodii DSM 1030* | Awo_RS_04145-04180 |
| *Cloacibacillus porcorum strain CL-84^1^* | BED41_RS16035-00320 |
| *Clostridium beijerinckii strain NCIMB 14988^2^* | LF65_RS20925-20895 |
| *Clostridium carboxidivorans P7^1^* | Ccar_RS16075-16045 |
| *Clostridium difficile 630* | CD630_33170-33130 |
| *Clostridium taeniosporum strain 1/k^2^* | BGI42_RS14085-14055 |
| *Desulfotalea psychrophila LSv54* | DP_RS02385-02370 |
| *Desulfobacterium autotrophicum HRM2* | HRM2_RS00765-00750 |
| *Desulfotomaculum nigrificans CO-1-SRB^3^* | DESCA_RS05360-14360 |
| *Desulfovibrio alaskensis G20* | DDE_RS02105-02120 |
| *Desulfovibrio magneticus RS-1* | DMR_RS20510-20490 |
| *Desulfovibrio salexigens DSM 2638* | DESAL_RS01825-01840 |
| *Dehalobacterium formicoaceticum strain DMC^4^* | CEQ75_RS07685-07715 |
| *Ilyobacter polytropus DSM 2926*^5^ | ILYOP_RS15475-03270 |
| *Intestinibacter bartlettii DSM 16795* | CLOBAR_00271-00268 |
| *Paenibacillus durus strain DSM 1735* | PDUR_RS13050-13065 |
| *Paenibacillus polymyxa SC2* | PPSC2_RS42955-42970 |
| *Paenibacillus stellifer strain DSM 14472* | PSTEL_RS12765-12775 |
| *Paenibacillus terrae HPL-003* | HPL003_23040- 23055 |
| *Pelosinus fermentans JBW45* | JBW_RS18290-18275 |
| *Peptoclostridium acidaminophilum DSM 3953*^6^ | EAL2_RS02100-15100 |
| *Symbiobacterium thermophilum IAM 14863* | STH_RS16410-16395 |
| *Thermoanaerobacter kivui strain DSM 2030* | TKV_RS09735-09720 |
| *Thermosediminibacter oceani DSM 16646^5^* | TOOE_RS03915-03930 |
| *Treponema primitia ZAS-2* | TREPR_RS04760-04785 |

^1^genes annotated as *nuoE* and *nuoF* (NADH:Quinone oxidoreductase) between *hydA* and *fdhF*

^2^genes for molybdopterin biosynthesis and *fdhD* between *hydA* and *fdhF*

^3^no hyc genes, gene annotated as NAD/FAD oxidoreductase with one predicted transmembrane helix after fdhF

^4^genes annotated as *nuoE* and *nuoF* (NADH:Quinone oxidoreductase) in front of *hydA* and *fdhF*

^5^ no hyc genes

^6^genes annotated as *nuoE* and *nuoF* in front of *hydA*, genes annotated as NADH dehydrogenase in front of *fdhF*

Table S2. Comparison of purified enzymes catalysing the direct hydrogenation of CO_2_ as well as formate oxidation.

|  |  | H_2_:MV-oxidoreductase  activity [U/mg] | formate:MV-  oxidoreductase  activity [U/mg] | K_m_  (H_2_/ formate)  [mM] | H_2_ production  [U/mg]/[h^-1^] | formate  production  [U/mg] ]/[h^-1^] |
| --- | --- | --- | --- | --- | --- | --- |
| ***T. kivui*** | **60 °C** | 14,400 | 455 | 0.126/  0.55 | 930/  9,892,000 | 900/  9,556,000 |
| ***A. woodii^1^*** | **30 °C** | 10,800 | 600 | 0.125/  1.0 | 14/  141,960 | 10/  101,600 |
| ***E. coli^2,3,4^*** | **37 °C** | 2.44 ± 0.62^#^ | 0.82 ± 0.04^#^ | 0.034/  26 | 0.1/  1,836 | 0.03/  550.8 |

^#^ For *E. coli* FHL, benzylviologen instead of methylviologen was used

^1^ Schuchmann K, Müller V. Direct and reversible hydrogenation of CO_2_ to formate by a bacterial carbon dioxide
 reductase. Science. 2013;342:1382-5.
^2^ McDowall JS, Murphy BJ, Haumann M, Palmer T, Armstrong FA, Sargent F. Bacterial formate hydrogenlyase
 complex. Proc Natl Acad Sci USA. 2014;111:E3948-E56.
^3^ Axley MJ, Grahame DA. Kinetics for formate dehydrogenase of *Escherichia coli* formate-hydrogenlyase.
 J Biol Chem. 1991;266:13731-13736.
^4^ Pinske C, Sargent F. Exploring the directionality of *Escherichia coli* formate hydrogenlyase: a membrane-
 bound enzyme capable of fixing carbon dioxide to organic acid. MicrobiologyOpen. 2016;5:721-37.
ND – not determined

**
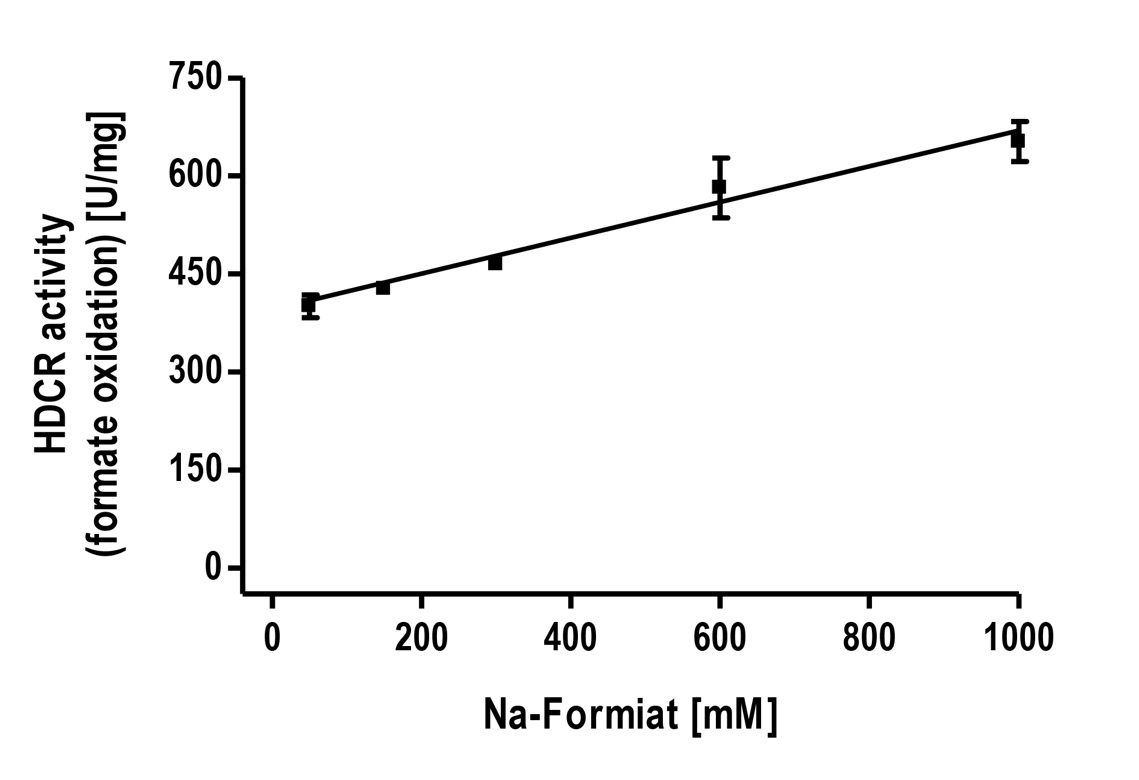
**

Figure S1. Substrate tolerance of the HDCR from *T. kivui*. 10 µg isolated enzyme was incubated in 100 mM HEPES/NaOH, 2 mM DTE, pH 7.0 under an atmosphere of 100% N_2_ and varying concentrations of Na formate as substrate. H_2_ production was measured in the gas phase.

**
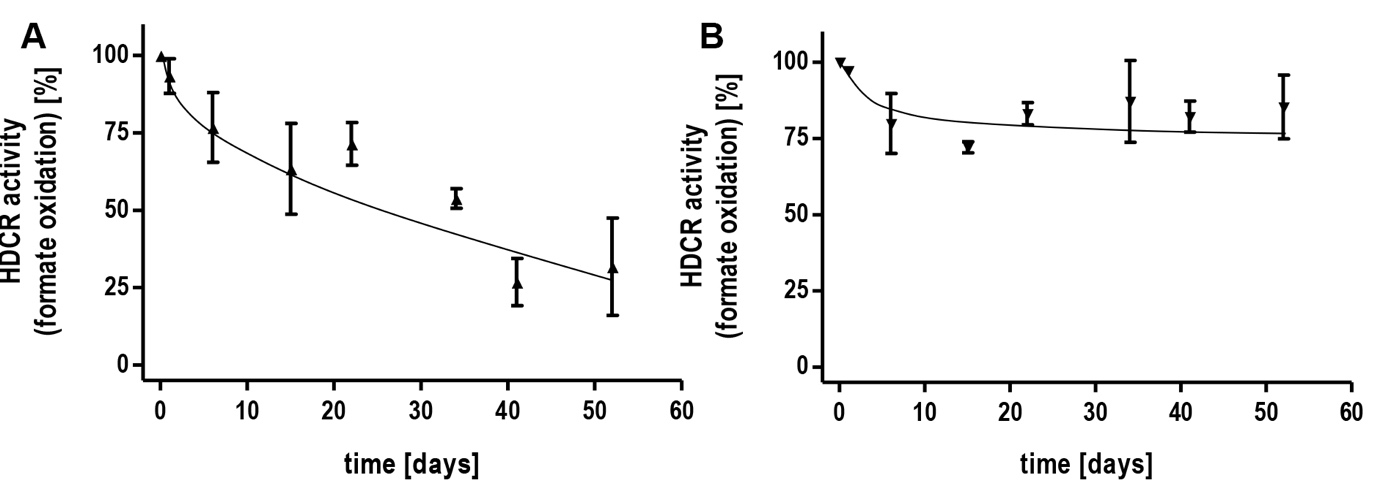
**

Figure S2. Stability of the HDCR from *T. kivui*. The enzyme was incubated at 4°C (A) and -20°C (B). Samples were taken and the specific activity determined by measuring H_2_ evolution. 10 µg isolated enzyme was incubated in 100 mM HEPES/NaOH, 2 mM DTE, pH 7.0 under an atmosphere of 100% N_2_ and 150 mM sodium formate as substrate. H_2_ production was measured in the gas phase. The sample incubated at -20°C was thawed each time and frozen afterwards.
